# Antigen stasis and airway nitrosative stress in human primary ciliary dyskinesia

**DOI:** 10.1152/ajplung.00208.2022

**Published:** 2024-02-06

**Authors:** Benjamin Gaston, Laura A. Smith, Michael D. Davis, Jessica Saunders, Ivana Daniels, Amjad Horani, Steven L. Brody, Olivia Giddings, Yi Zhao, Nadzeya Marozkina

**Affiliations:** ^1^Herman B. Wells Center for Pediatric Research, Riley Hospital for Children, Indiana University School of Medicine, Indianapolis, Indiana, United States; ^2^Department of Medicine, Washington University, St. Louis, Missouri, United States; ^3^Department of Medicine, Case Western Reserve University, Cleveland, Ohio, United States; ^4^Department of Biostatistics and Health Data Science, Indiana University School of Medicine, Indianapolis, Indiana, United States

**Keywords:** DUOX1, nasal NO, nitrosative stress, oxidation, primary ciliary dyskinesia

## Abstract

Nasal nitric oxide (nNO) is low in most patients with primary ciliary dyskinesia (PCD). Decreased ciliary motion could lead to antigen stasis, increasing oxidant production and NO oxidation in the airways. This could both decrease gas phase NO and increase nitrosative stress. We studied primary airway epithelial cells from healthy controls (HCs) and patients with PCD with several different genotypes. We measured antigen clearance in fenestrated membranes exposed apically to the fluorescently labeled antigen *Dermatophagoides pteronyssinus* (Derp1-f). We immunoblotted for 3-nitrotyrosine (3-NT) and for oxidative response enzymes. We measured headspace NO above primary airway cells without and with a PCD-causing genotype. We measured nNO and exhaled breath condensate (EBC) H_2_O_2_ in vivo. Apical Derp1-f was cleared from HC better than from PCD cells. DUOX1 expression was lower in HC than in PCD cells at baseline and after 24-h Derp1-f exposure. HC cells had less 3-NT and NO_3_^−^ than PCD cells. However, NO consumption by HC cells was less than that by PCD cells; NO loss was prevented by superoxide dismutase (SOD) and by apocynin. nNO was higher in HCs than in patients with PCD. EBC H_2_O_2_ was lower in HC than in patients with PCD. The PCD airway epithelium does not optimally clear antigens and is subject to oxidative and nitrosative stress. Oxidation associated with antigen stasis could represent a therapeutic target in PCD, one with convenient monitoring biomarkers.

**NEW & NOTEWORTHY** The PCD airway epithelium does not optimally clear antigens, and antigen exposure can lead to NO oxidation and nitrosative stress. Oxidation caused by antigen stasis could represent a therapeutic target in PCD, and there are convenient monitoring biomarkers.

## Graphical abstract

**Figure:**
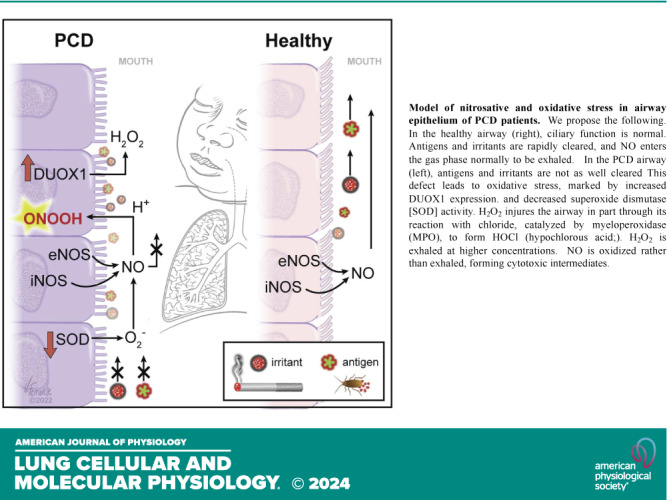

## INTRODUCTION

Primary ciliary dyskinesia (PCD) is caused by pathological variants in many different genes that affect the formation, structure, and function of motile cilia (1). PCD may be more common than was previously thought, with a worldwide incidence estimated to be at least one in 7,600 (2). The diagnosis of PCD is challenging, and measurement of nasal nitric oxide (nNO) has been widely adopted as a biomarker for disease diagnosis in individuals with features typical of PCD (3–5). These features include newborn respiratory distress; chronic nasal and sinus disease; chronic cough, bronchitis, and bronchiectasis; and disorders of left-right symmetry (1*–*5). Although most patients with a confirmed diagnosis of PCD have low nNO (3–5), the reasons for this finding are unclear.

Expression of NO synthase (NOS) isoforms is not consistently different in PCD airways compared with healthy individuals (5; see also Supplemental Fig. S1). With regard to NOS activity, we have previously reported that decreased ciliary motion prevents airway epithelial endothelial NOS (eNOS) activity, and this difference in activity could contribute to low airway NO values (6). However, this phenomenon may not fully explain the profoundly low nNO values observed in most patients with PCD. An additional potential explanation for low nNO is that NO could be oxidized before leaving the sinuses and nose (7, 8). This possibility is strengthened by recent evidence that antigens on the airway epithelial surface may increase the expression and/or activity of pro-oxidant enzymes in airway epithelial cells (9–11). NO reacts with oxidants, including O_2_^−^, O_2_, and H_2_O_2_; of these, reactions with O_2_^−^ are by far the most rapid (7, 8, 12, 13). Kinetics vary widely and, in some cases, are not first order (i.e., accelerate exponentially at higher reactant concentrations). Products include NO_2_, HNO_2_/NO_2_^−^, NO_3_^−^, HONOO/ONOO^−^, and other aqueous nitrogen oxides (6–8, 12, 13). All of these reactions deplete the reactant, NO. Depending on conditions such as pH, the products can have cytotoxicities (7, 8, 13). One pro-oxidant enzyme is dual oxidase 1 (DUOX1), upregulation of which during antigen stasis is mediated by P2Y and PAR receptors, leading to the activation of type 2 alarmins (14, 15). Upregulation of DUOX1 activity may also directly affect ciliary activity (10). P2Y and PAR activation have been linked with activation of *DUOX1*, but not necessarily with upregulation of DUOX1 protein levels. Activity and/or expression of other oxidative enzymes, including NADPH oxidases (NOXs), could also be upregulated, and airway irritants also inhibit the activity of antioxidant systems such as superoxide dismutase (SOD) (16, 17). We hypothesized that PCD airway cells might not efficiently clear antigens from the epithelial surface and that this defect would increase oxidative stress. This oxidative stress, in turn, could both decrease gas phase NO concentrations and contribute to airway nitrosative stress and airway epithelial injury.

## MATERIALS AND METHODS

### Human Studies

Studies involving human subjects were reviewed and approved by the relevant institutional review boards (IRBs). Specifically, nNO measurement was approved by University Hospitals (UHs), Cleveland, Protocol No. 07-13-30, breath H_2_O_2_ measurement by Indiana University Purdue University Indianapolis (IUPUI) Protocol No. 1910580775, nasal brush for epithelial cell culture by UH Cleveland Protocol No. 10-04-14, and nasal brush for epithelial cell culture by IUPUI Protocol No. 1408855616. Written informed consent was obtained from all subjects for these procedures.

### Materials

Materials were purchased from Sigma-Aldrich (St. Louis, MO) unless otherwise noted.

### Primary Cell Culture

Primary healthy control (HC) human airway epithelial cells were grown at the air-liquid interface (ALI) on Transwell filters (Corning, 3460; Corning, NY) from nasal brushings and from explanted lung tissue as previously described (6, 9). Nasal brushes were obtained from nonsmoking HCs and from subjects with PCD in our PCD clinics, and normal bronchial cells were purchased commercially (Lifeline Cell Technologies, Frederick, MD). Subjects with PCD had the following genotypes: two disease-causing alleles in trans for *1*) *CCNO* (consanguineous:homozygous for C807T > A), *2*) *CCDC40* (248delC and 3097 A > T), *3*) *DNAH11* (4453 C > T and 13331 G > C), and *4*) *CCDC39* (831delCA and 1872delTA). Cells from these subjects were used when early passage was available; not all cell lines were available for all experiments. Cells were grown at ALI until fully differentiated as pseudostratified epithelial cells (∼6 wk) as previously described (6, 9). The specific PCD genotypes were chosen both because they represented heterogeneous pathogenic mechanisms and because patients with these genotypes were available for participation. All cells except those from the *CCNO* patient developed abundant cilia (18). In some experiments, cells were incubated with or without 3,000 units of Cu/Zn superoxide dismutase (SOD) (19) or 300 μM apocynin (20, 21).

### Antigen Clearance Assay

**Figure 1.**
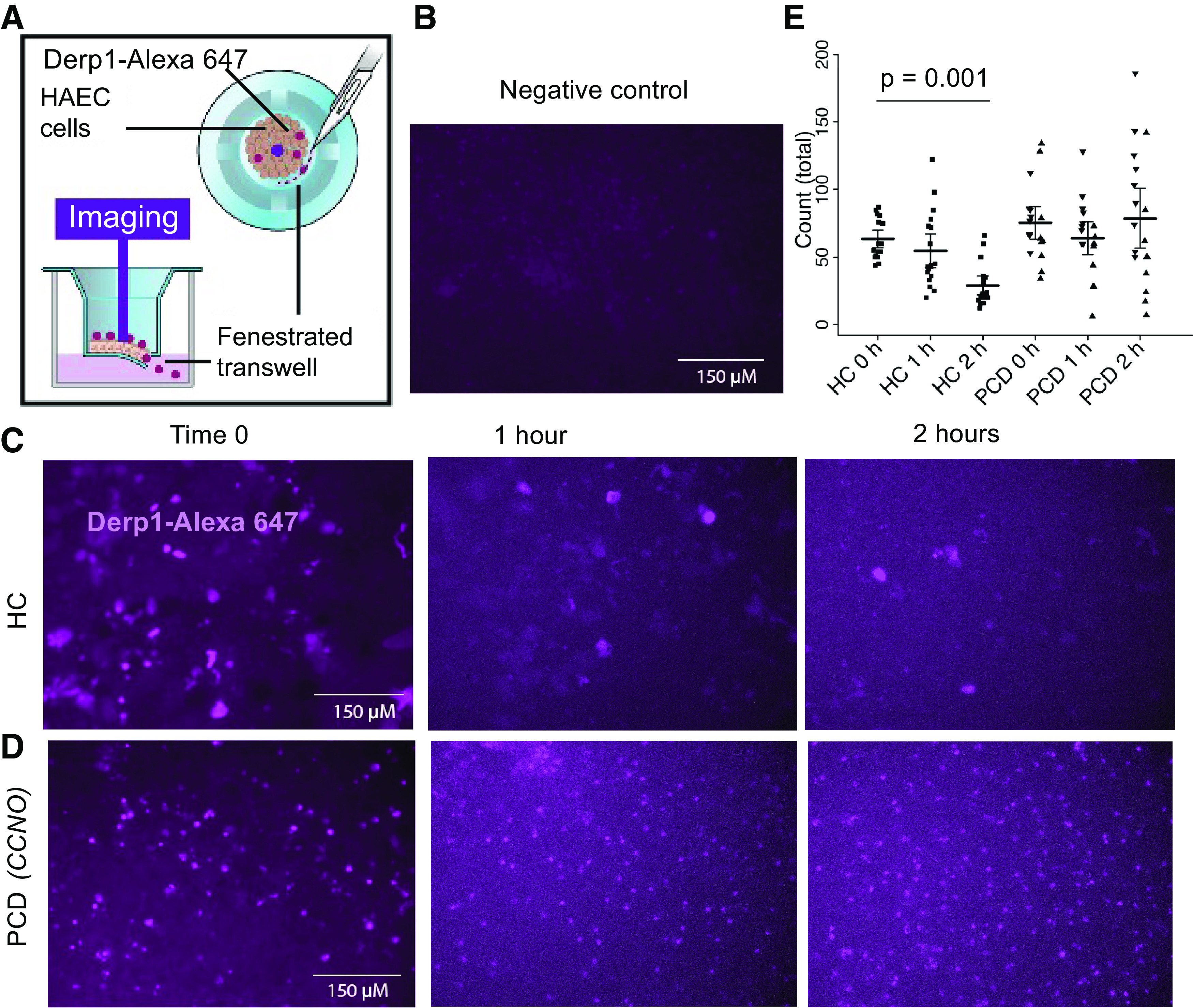
Clearance of fluorescently labeled Derp1 from the apical surface of ciliated HC and PCD human airway epithelial cells. *A*: in our model, cells (HC and PCD) were grown on Transwell filters until fully ciliated (4–6) wk. A fenestration was then cut circumferentially in the filter in a 90° arc, such that the apical (ciliated) surface was in contact with the basolateral medium. Fluorescently labeled Derp1 (Alexa-Fluor 647) was applied to the cell surface, and fluorescent images were obtained from the center of the field obtained at times 0, 1, and 2 h. Counts of labeled Derp1-f were made by three blinded observers. *B*: negative control (no Derp1-f added) shows essentially no background autofluorescence. *C* and *D*: representative images of labeled Derp1-f on the surface of HAECs: HC (*C*) and PCD (*D*) cells (*CCNO* genotype). *E*: quantitation of Derp1-f in cells from HC (*n* = 3 filters each from five subjects) and patients with PCD (*CCNO*, *n* = 9 filters and *DNAH11*, *n* = 6 filters). Comparisons were conducted via the generalized estimation equation (GEE) and comparison *P* values were corrected following Tukey’s procedure. □, HC cells; △, PCD *DNAH11* cells; and ▿, PCD *CCNO* cells. In HC cells, Derp1-f label decreased, comparing time 0 with 2 h. In PCD cells, there was no difference in signal between time 0, 1, and 2 h, neither for PCD as a whole nor for *CCNO* or *DNAH11*. Bars in *C* and *D* = 150 µm. HC, healthy control; PCD, primary ciliary dyskinesia.

*Dermatophagoides pteronyssinus* allergen 1 (Derp1) was purchased from Indoor Biotechnologies (Charlottesville, VA; RP-DP1D-1) and conjugated to Alexa Fluor 647 using the manufacturer’s protocol (ThermoFisher Scientific; A30009). HCs and PCD cells were grown on filters for 6 wk. In some experiments, filters were fenestrated circumferentially using a scalpel to produce a continuous opening along the edge of the membrane along a 90° arc (Fig. 1*A*), allowing a lip of membrane to hang into the medium and permitting direct communication between the ciliated cells on top of the filter and the basolateral medium. Cells were then exposed apically to fluorescently labeled Dep1 (Derp1-f), which was imaged in the center of the filter using an EVOS M5000 microscope at time 0, 1, and 2 h. Fluorescent labels from photomicrographs were counted by three blinded observers (Fig. 1*E*).

### Immunoblots

#### 3-Nitrotyrosine.

3-Nitrotyrosine (3-NT) proteins from cell lysates (1.5 μg total) were separated on the ProteinSimple Jess Capillary electrophoresis system. To detect 3-nitrotyrosine, whole cell lysates from HCs and *DNAH11* cells were immunoblotted for 3-nitrotyrosine, a stable protein modification caused by tyrosine nitration (13), using rabbit antinitrotyrosine antibody (Cell Signaling, Cat. No. 9691, 1:10 in ProteinSimple Milk-Free Antibody Diluent). Levels were normalized to β-actin using rabbit anti-β-actin antibody (Cell Signaling, Cat. No. 3700, 1:300 ProteinSimple Milk-Free Antibody Diluent). Secondary antibodies were purchased from ProetinSimple (anti-rabbit, DM-001 and anti-mouse, DM-002). Blots were visualized and analyzed using the ProteinSimple Compass for SW software.

#### DUOX1, NOX1, NOX2, NOX3, NOX4, iNOS, and xanthine oxidase protein expression.

Expression was measured using the ProteinSimple Jess capillary electrophoreses system (San Jose, CA). Cell lysate (1.5 µg) was separated according to the manufacturer’s protocol, and DUOX1 was detected using a primary goat anti-DUOX1 antibody (OriGene, Cat. No. TA320203, 1:50) and an anti-goat secondary antibody (ProteinSimple Cat. No.: DM-006). DUOX1 levels were normalized to β-actin using rabbit anti-β-actin antibody (Cell Signaling, Cat. No. 3700, 1:300). NOX1 and NOX3 protein expression were measured using the ProteinSimple Jess capillary electrophoreses system (San Jose, CA). Cell lysate (1.5 µg) was separated according to the manufacturer’s protocol, and NOX1 and NOX3 were detected using a primary rabbit anti-NOX1 antibody (Proteintech Group, Cat. No. 17772-1-AP, 1:50), a NOX3 antibody (Proteintech Group, Cat. No. 20065-1-AP, 1:25), and an anti-rabbit secondary antibody (ProteinSimple, DM-001). NOX1 and NOX3 levels were normalized to mouse anti-β-actin (Cell Signaling, Cat. No. 3700, 1:300) and anti-mouse-NIR secondary antibody (ProteinSimple DM-009). iNOS was detected using a primary goat anti-iNOS antibody (R&D, Cat. No. MAB9502, 1:20) and an anti-goat secondary antibody (ProteinSimple, DM-006). iNOS levels were normalized to mouse anti-β-actin (as above). NOX2 was detected using a primary Rabbit anti-NOX2 antibody (Proteintech Group, 19013-1-AP, 1:100), NOX4 antibody (Proteintech Group, 14347-1-AP, 1:200), and an anti-rabbit secondary antibody (ProteinSimple, DM-001); NOX2 and NOX4 levels were normalized to mouse anti-β-actin. Xanthine oxidase (XO) was detected using a primary rabbit anti-XO antibody (Proteintech Group, 55156-1-AP, 1:200) and an anti-rabbit secondary antibody (ProteinSimple, DM-001); XO levels were normalized to mouse anti-β-actin. 

### Nitrate Assay

Nitrate (NO_3_^−^) accumulation in media was measured colorimetrically after reduction by vanadium chloride as previously described (7, 22–24).

### Superoxide Assay

Superoxide was measured in HC and PCD *DNAH5* cells according to the manufacturer’s protocol (Enzo ROS-ID Superoxide Detection Kit, Cat. No. ENZ-51012). Cells were grown on transwells as described earlier, then loaded with a superoxide staining solution, and incubated for 1 h. Untreated cells served as a control. After 1 h of incubation, cells were washed in the washing buffer and observed under a fluorescent microscope (EVOS M5000) using standard excitation/emission filter sets compatible with Rhodamine (Ex/Em 550/620 nm). Correlated total cell fluorescence (CTCF) was calculated as a difference between Integrated density − (Area of selected cell × Mean fluorescence of background readings) using ImageJ.

### Headspace NO Assay

Cells from HC or from subjects with PCD (*DNAH11*, *CCNO*) were placed in media in an 8-mL glass vial with a sealed membrane cap containing 1 mL of medium, one membrane per vial, with or without 3,000 units of SOD or 300 μM of the NOX inhibitor, apocynin (20). NO (1 mL of 45 ppm diluted 1:1,000; Gasco, Noblesville, IN; x02NI99CP160039) was injected into the sealed gas compartment above the cells and medium (the headspace), and the sample was gently vortexed (5 s) and then incubated at 37°C. Samples for gas analysis were withdrawn at time 0 and at 30 min. NO was measured by a Sievers NOA 280i analyzer (Zysense, Waddington, NC). We measured the first peak (the effect of the plunger at the bottom of the syringe at the end of the injection generated a second, artifactual, peak). We compared peaks between time 0 (*t*_0_) and time 30 min (*t*_30 min_).

### Nasal nNO Assay

Nasal nNO was measured as described previously (3, 4) in 7 HC volunteers and 12 subjects who met clinical criteria for PCD (3) using a Sievers NOA 280i (3).

### Exhaled Breath Condensate Collection and Analysis

H_2_O_2_ levels in exhaled breath condensate (EBC) were measured from five healthy HCs and from five subjects who met clinical criteria for PCD (3) using the Inflammacheck point-of-care device (Exhalation Technologies, Cambridge, UK) according to the manufacturer’s recommendations. Collection was performed in accordance with the European Respiratory Society/American Thoracic Society technical standards (25).

### Statistical Analysis

For data with a Gaussian distribution, we used the Student’s unpaired, two-tailed *t* test. For analyses with more than two groups, robust regression was used and corresponding contrasts were established for comparison. For non-Gaussian data sets, as determined by the Shapiro–Wilk test, we used the Wilcoxon rank-sum test. For repeated measures, we used a generalized estimating equation (GEE) to conduct obust comparison between groups and measurements. Multiple testing corrections were performed following Tukey’s procedure when considering pair-wise comparisons and the Bonferroni correction for the rest.

## RESULTS

### Antigen Stasis on PCD Epithelial Cells

To assess antigen stasis on the PCD epithelium, we studied the loss of Derp1-f from the surface of airway epithelial cells from HC and subjects with PCD cultured at ALI. We used Derp1 allergen in our study because it belongs to *group 1* allergens, and at least 70% of allergic individuals recognize *group 1* allergens. Two sets of PCD disease-causing variants were used for these studies: *1*) *DNAH11*, which results in cilia with ineffective beating (26), and *2*) *CCNO,* which results in reduced generation of motile cilia (18). The Transwell filters were fenestrated to allow the movement of Derp1-f from the surface to the basal compartment (Fig. 1*A*). In cell preparations from HCs, a mean of 59 ± 7% of Derp1 was cleared from the apical surface of the epithelium after 2 h (*n* = 3 filters each from five subjects, *P* = 0.001 compared with time 0) (Fig. 1, *B–E*). Derp1-f was poorly cleared from filters with either *DNAH11* or *CCNO* genotype; the antigen clearance was 27 ± 26% (*n* = 6 filters from one subject with *DNAH11*, *n* = 9 filters from one subject with *CCNO*; *P* = NS comparing time 0 with 2 h) (Fig. 1, *B–E*). The antigen clearance at 2 h in HC cells was significantly greater than in PCD cells (*P* = 0.001). The study was not extended beyond 2 h as the baseline fluorescent signal became attenuated in all cells after 2 h.

### Upregulation of DUOX1 in PCD Airway Epithelial Cells at Baseline and with Derp1 Stasis

**Figure 2.**
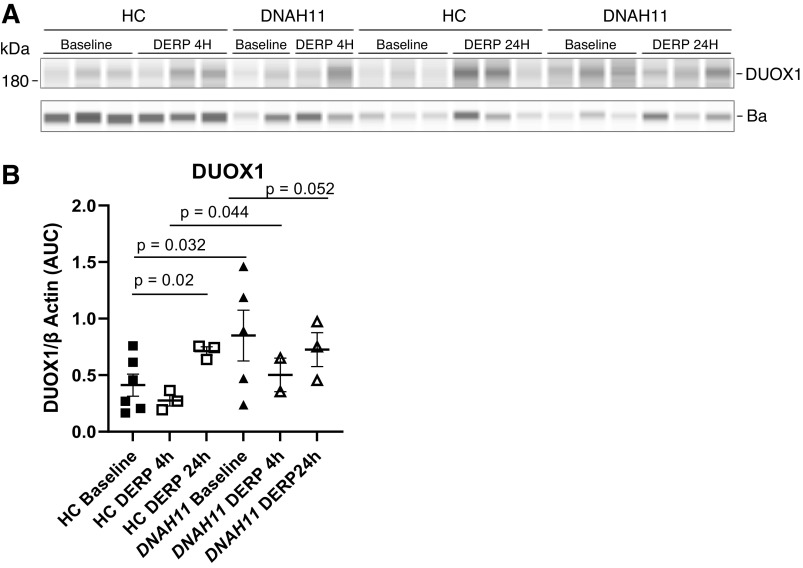
DUOX1 expression and superoxide production in PCD cells. *A*: immunoblots of DUOX1 expression in HC cells and PCD (*DNAH11*) cells grown at ALI at baseline and after 4 and 24 h Derp1 exposure (*n* = 3 each independent filters). *B*: quantitation of DUOX1 densities. Derp1 increased DUOX1 expression in HC cells after 24 h. Comparisons were conducted via the generalized estimation equation (GEE) and comparison *P* values were corrected following Tukey’s procedure. □, HC cells; △, PCD *DNAH11* cells. In the aggregate, the DUOX1 expression in PCD (*DNAH11*) cells at ALI was elevated at baseline relative to HC (*n* = 3 each). Both at 4 and 24 h, the increased values of DUOX1 after Derp1 exposure were somewhat higher in PCD cells than those after a similar exposure to HC cells. HC, healthy control; PCD, primary ciliary dyskinesia.

Consistent with previous data (14, 15), Derp1 increased DUOX1 expression in relation to β-actin in HC cells at ALI after 24 h exposure, from 0.45 ± 0.004 to 0.83 ± 0.14 densitometry units/protein densitometry (*n* = 3 each; *P* = 0.02, control, time 0 compared with 24 h), (Fig. 2, *A* and *B*). DUOX1 expression in PCD (*DNAH11*) cells was also elevated at baseline (0.61 ± 0.016) relative to that in HC cells (*n* = 3 each; *P* = 0.032, at time 0). Compared with time 0 h, at 24 h, DUOX1 increased in PCD (*DNAH11*) cells (from 0.61 ± 0.016 to 1.36 ± 0.17 densitometry units at 24 h) following exposure to Derp1. Taken together, these data suggest that elevated DUOX1 expression in the airway epithelium of patients with PCD may, at least in part, be the result of an inability to efficiently clear antigens from its surface.

### Upregulation of Superoxide Production in PCD Cells

Superoxide was assayed using the fluorescent assay as described in materials and methods. Levels were higher in PCD cells (three technical replicates each from two patients with *DNAH5* 256 ± 53 × 10^6^ CTCF) than in HC cells (172 ± 11 × 10^6^ CTCF) by unpaired *t* test (*P* = 0.019). Both values were significantly greater than the negative control (Fig. 2*C*). However, we did not find a consistent upregulation of NOX 1–4 or of XO protein expression in PCD cells. (Supplemental Figs. S2–S4). Taken together, these data suggest that oxidant production may be upregulated in PCD by changes in enzyme activity more than by changes in protein expression. For reasons that are unclear, NOX2 protein expression was actually lower in HC cells after 24 h exposure to Derp1 (Supplemental Fig. S3).

### Loss of NO in PCD Cells

**Figure 3.**
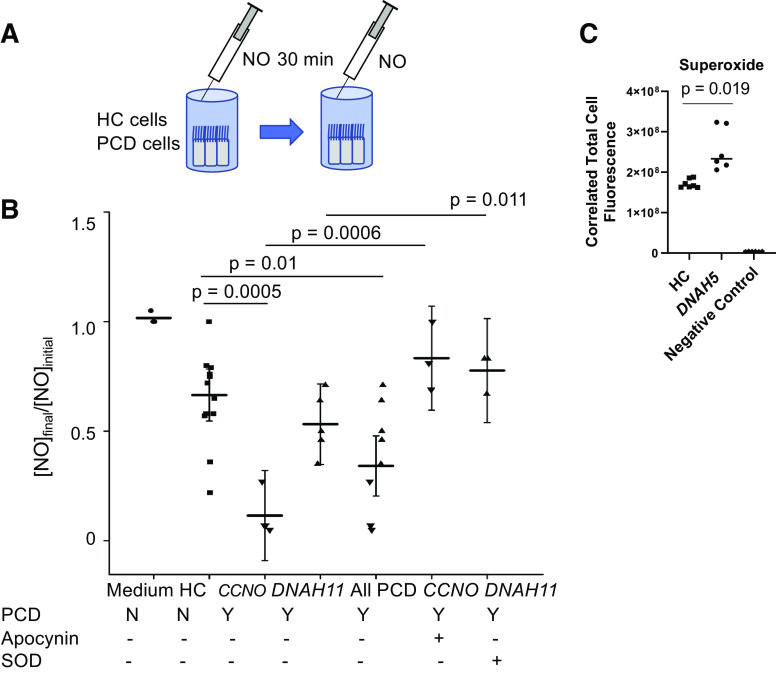
Reduced NO levels in PCD cells. Headspace NO was measured in cells from HC and subjects with PCD at baseline and after 30 min. *A*: in our model, NO was injected through a septum into sealed vials; initial and final NO concentrations were then measured from the gas over the cells (Headspace). *B*: the ratio of final/initial headspace NO lost over PCD cells (0.12 ± 0.10, *n* = 3 PCD *CCNO* and 0.532 ± 0.14, *n* = 5 PCD *DNAH11*) was lower than that over HC airway cells (0.65 ± 0.20, *n* = 9 controls, three each from three subjects). Compared with no treatment, treating PCD *DNAH11* cells with the antioxidant enzyme SOD (*n* = 3) increased headspace NO final to initial ratio to 0.78 ± 0.092. Treatment with apocynin (NADPH oxidase inhibitor) increased headspace NO in PCD cells (*CCNO;* 0.83 ± 0.15, *n* = 3). Comparisons were conducted via robust regression and comparison *P* values were corrected following Tukey’s procedure. ○, cell culture medium; □, HC cells; △, PCD *DNAH11* cells; ▿, PCD *CCNO* cells. *C*: superoxide was measured in HC (*n* = 9) and PCD *DNAH5* cells (*n* = 9) using the fluorescent assay as described in materials and methods with a fluorescent microscope equipped with standard excitation/emission filter sets comparable with Rhodamine (Ex/Em 550/620 nm). Negative control cells were treated/untreated with the fluorescent probe (autofluorescence; *n* = 6). CTCF was calculated as described in materials and methods using ImageJ. □, HC cells; ○, PCD *DNAH5* cells; ◊, negative control (Control), autofluorescence. Superoxide levels were higher in both HC and PCD than in negative control and were lower in HC than in PCD. CTCF, correlated total cell fluorescence; HC, healthy control; NO, nitric oxide; PCD, primary ciliary dyskinesia; SOD, superoxide dismutase.

Loss of NO in the epithelium was assessed as the ratio of final to initial headspace NO collected in sealed glass chambers containing filters of mature ALI cultures and medium (see Fig. 3*A*). The ratio of final/initial headspace NO lost over PCD cells (0.115 ± 0.10, *n* = 3 PCD *CCNO* and 0.532 ± 0.14, *n* = 3 PCD *DNAH11*) was lower than that over HC airway cells (0.648 ± 0.20; *n* = 9 controls, three from each subject; *P* = 0.0005). Compared with no treatment, treating PCD *DNAH11* cells with the antioxidant enzyme SOD increased headspace NO final to initial ratio to 0.776 ± 0.092 (*P* = NS; Fig. 3*B*). Treatment with apocynin (NADPH oxidase inhibitor) increased headspace NO in HC airway cells (0.898 ± 0.11, *n* = 5, *P* = NS; HC vs. HC + apocynin) and in PCD cells (*CCNO*; 0.833 ± 0.15, *n* = 3, *P* = 0.0006; PCD untreated vs. PCD + apocynin). Inducible NOS protein expression was not lower in PCD cells; in fact, it was higher after Derp1 exposure (Supplemental Fig. S1).

### NO Oxidation Products in PCD Cells

**Figure 4.**
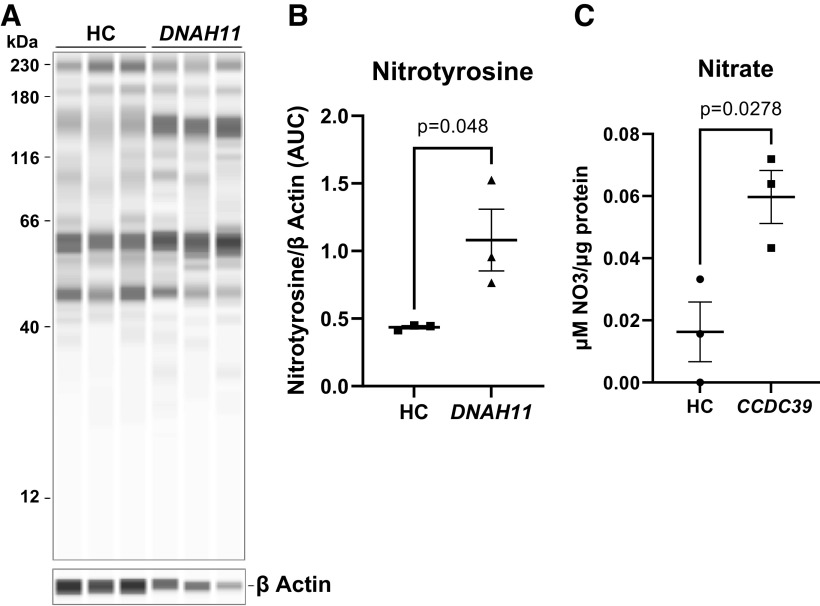
Nitrotyrosine and nitrate are increased in PCD cells. Lysates for cells from HC and subjects with PCD were immunoblotted for the superoxide footprint, tyrosine nitration. *A*: immunoblots of protein 3-nitrotyrosine immunostaining in HC and PCD (*DNAH11*) cells. *B*: densitometry and quantitation of *A* normalized to β-actin (*n* = 3 filters from each cell type). □, HC cells; △, PCD *DNAH11* cells. *C*: superoxide and NO produce ONOO^−^/NO_3_^−^. Concentrations of the NO oxidation product NO_3_^−^ in PCD (*CCDC39*) media (*n* = 3) were higher than in HC media (*n* = 3). ○, HC cells; □, PCD *CCDC39* cells. Comparisons were conducted via unpaired *t* test. HC, healthy control; NO, nitric oxide; PCD, primary ciliary dyskinesia.

We next studied the cellular “footprint” of NO oxidation, protein 3-nitrotyrosine (3-NT) immunostaining, in HC and PCD (*DNAH11*) cells at ALI, with and without iNOS upregulation (13, 22). At baseline, HC immunostaining for 3-NT was less in HC cells than in PCD cells (*n* = 3 each; *P* = 0.048) (Fig. 4, *A* and *B*) (22). Superoxide and NO produce ONOO^−^/NO_3_^−^. Concentrations of the NO oxidation product NO_3_^−^ were higher in PCD (*CCDC39*) media (0.06 ± 0.014 μM/μg protein) than in HC media (0.017 ± 0.016 μM/μg protein), *P* = 0.027 (Fig. 4*C*). Taken together, these findings suggest that NO is oxidized, primarily by superoxide, in the PCD airway epithelium relative to control airway epithelium.

### In Vivo Measurements of Airway Oxidation and of Gas Phase NO

**Figure 5.**
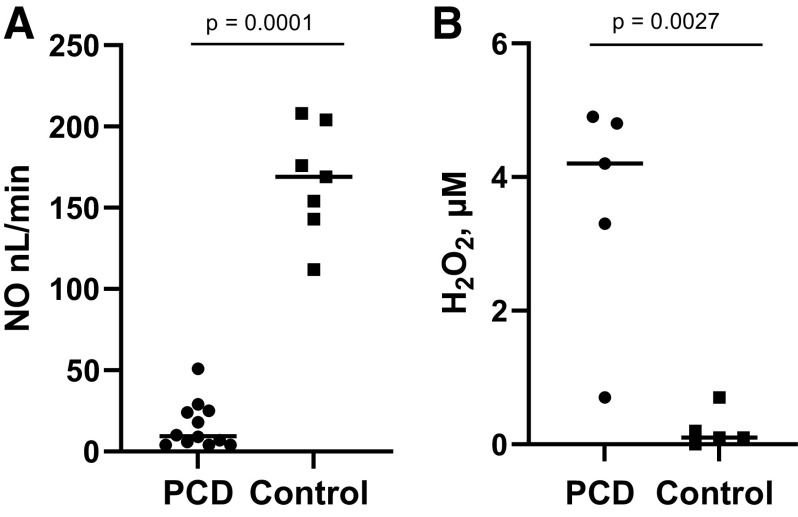
Nasal NO production and breath H_2_O_2_ production in healthy control and subjects with PCD. *A*: nasal NO production was measured using the standard PCD Foundation protocol by NOA 280i in HC and subjects with PCD. Nasal NO production was higher in HC (*n* = 7) than in PCD (*n* = 12). *B*: exhaled breath condensate H_2_O_2_ production in HC was lower than in subjects with PCD (*n* = 5 each). In both *A* and *B*, comparisons were conducted via unpaired *t* test. □, HC; ○, PCD. HC, healthy control; NO, nitric oxide; PCD, primary ciliary dyskinesia.

**Figure 6.**
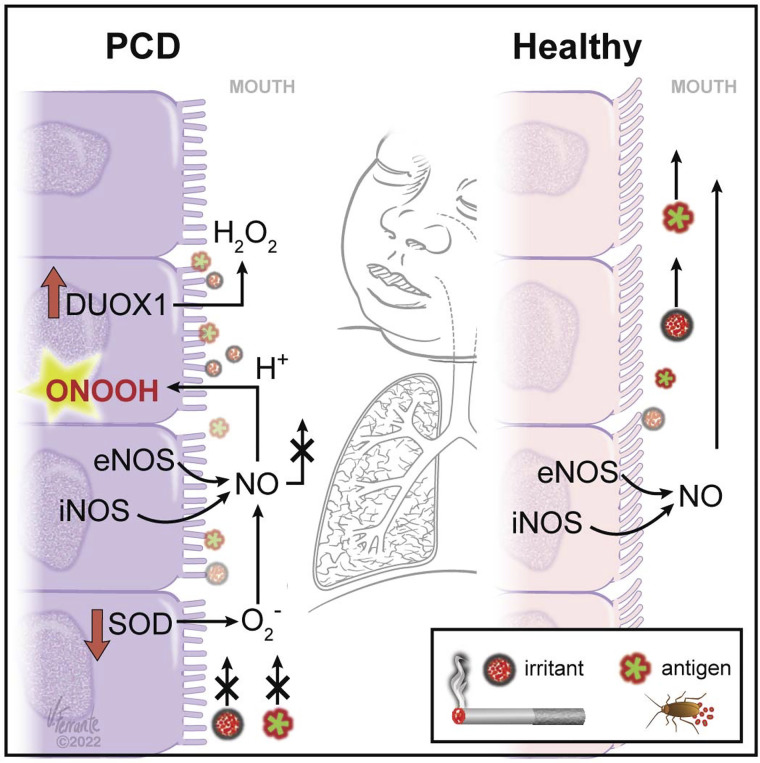
Model of nitrosative and oxidative stress in airway epithelium of patients with PCD. We propose the following. In the healthy airway (*right*), ciliary function is normal. Antigens and irritants are rapidly cleared, and NO enters the gas phase normally to be exhaled. In the PCD airway (*left*), antigens and irritants are not as well cleared (Fig. 1). This defect leads to oxidative stress, marked by increased DUOX1 expression (Fig. 2; 12, 13) and decreased superoxide dismutase (SOD) activity (24). H_2_O_2_ injures the airway in part through its reaction with chloride, catalyzed by myeloperoxidase (MPO), to form HOCl (hypochlorous acid; 26). H_2_O_2_ is exhaled at higher concentrations (Fig. 5). NO is oxidized rather than exhaled, forming cytotoxic intermediates (Figs. 3, 4, and 5). eNOS, endothelial nitric oxide synthase; iNOS, inducible nitric oxide synthase; NO, nitric oxide; SOD, superoxide dismutase. [Ferrante Medical Media grants a nonexclusive license to American Physiological Society to use the image(s) in the *American Journal of Physiology-Lung Cellular and Molecular Physiology* electronically and in print indefinitely. Ferrante Medical Media will retain the copyright of the image.]

Confirming previous observations (3–5), we found that nNO was much higher in HC (167 ± 33 nL/min, *n* = 7) than in subjects with PCD (15.9 ± 14 nL/min, *n* = 12 PCD, *P* < 0.0001; Fig. 5*A*). Low NO (gas phase) levels in the setting of high concentrations of cellular/aqueous NO oxidation products are consistent with the loss of airway NO to oxidation in PCD (Fig. 6). Because DUOX1 generates H_2_O_2_, we next measured EBC for H_2_O_2_ in vivo. This was lower in HC than in subjects with PCD (0.22 ± 0.5 vs. 3.58 ± 2.5 µM vs., *n* = 5, *P* = 0.0027; Fig. 5*B*).

## DISCUSSION

Disease-causing variants in many different genes are associated with PCD (1, 2, 4, 18, 26, 27). Mutations in these genes cause several different defects in proteins, including defective synthesis, trafficking, formation, repair, structure, and coordinated function of cilia-related proteins. These all result in insufficient ciliary function, the defining feature of PCD. Most patients with disease-causing variants share common features, including chronic rhinitis and sinusitis, chronic cough, bronchiectasis, and low nNO levels (1, 3, 4). Our data suggest that low nNO in patients with PCD is, in part, caused by increased NO oxidation. Specifically, we show here that though gas phase NO is consumed more in the headspace above human airway cells grown from patients with PCD than from controls, the NO oxidation product, nitrotyrosine, is actually increased in PCD cells. Moreover, we show that particle clearance from the surface of cells in patients with PCD is impaired, and that this impairment can increase the expression and/or activity of oxidant enzymes, consistent with previous work (14, 15). We and others have also previously shown that irritant exposure decreases SOD activity in the airway (16), increasing superoxide levels. Although airway NO concentrations are low in PCD (1, 3–5, 29; Fig. 5), our evidence demonstrates that the oxidation products of NO in the PCD airway are high.

In addition to contributing to low NO concentrations in the PCD airway, oxidative stress could contribute to airway injury. Superoxide reacts rapidly with NO to form ONOO^–/^ONOOH (p*K*_a_ ∼6.5), and ONOOH reacts with protein tyrosines to cause protein injury through tyrosine nitration (7, 13). We show that this occurs in the PCD cells to a greater extent than in the HC cells (Fig. 4). Depending on conditions in the airways, additional oxidation products, such as HNO_2_/NO_2_^−^ can also be cytotoxic (7). Moreover, DUOX1 increases the generation of H_2_O_2_, which also leads to the formation of cytotoxic intermediates. We show here that breath H_2_O_2_ concentrations are increased in PCD. H_2_O_2_ injures the airway in part through its reaction with chloride, catalyzed by myeloperoxidase (MPO), to form hypochlorous acid (30). Immune cells, such as neutrophils, monocytes, and macrophages, are the main source of MPO in human airways. It can also injure the airway through a reaction with bromide, catalyzed by eosinophil peroxidase, to form hypobromous acid (31). Finally, increased DUOX1 expression can signal increased IL33 secretion, potentially initiating a Th2 cascade (32), though patients with PCD are not usually characterized as having generalized atopic symptoms, and, as noted earlier, they have low, as opposed to high, airway NO levels.

In addition to the formation of reactive oxygen species caused by antigen stasis, additional mechanisms may be relevant to the observation that NO is decreased in the PCD airway. For example, end-expired oxygen levels are higher in PCD epithelia than in normal subjects (33), or in subjects with cystic fibrosis (CF). The airways of subjects with CF have increased oxygen consumption compared with disease-free epithelium (34), and it may be that the PCD epithelium, by contrast, may have decreased metabolic needs and decreased oxygen consumption (33). Molecular oxygen reacts with NO to form cytotoxic NO_2_ radical (7); increased oxygen would also be expected to decrease NO. Furthermore, we have previously shown that eNOS activation—independently of eNOS expression—is regulated in part by the redox state of somatic cell Hb in the airway epithelium, and that loss of ciliary function decreases eNOS activity (35).

There are other potential reasons for decreased nNO in PCD, but none are compelling. Denitrifying organisms colonizing the airway lead to a modest (low ppb) decrease in airway NO in CF (36) and could contribute to decreased nNO in PCD, with the caveats that *1*) these organisms are generally less common colonizers in PCD than in CF (37), and *2*) airway NO levels are overall somewhat higher in CF than in PCD (5, 7). Airway pH can affect airway NO levels, but patients with PCD (unlike patients with CF) have no intrinsic reason to have low airway epithelial surface pH. Arginine, citrulline, and asymmetric dimethylarginine (ADMA) metabolism are not known to be disordered in patients with PCD, nor is *S*-nitrosothiol metabolism; these factors are unlikely to cause uniformly low nNO production rate values in PCD relative to HC or to other conditions (7).

The results of our study could prove to be clinically important for two reasons. First, they underscore the importance of airway clearance, exercise, and cough in moving antigens out of the PCD airway (38). Antigen stasis and oxidative/nitrosative stress appear likely to injure the airway epithelium. In fact, the efficacy of airway clearance over time might theoretically be monitored by changes in F_ENO_ (38). Second, patients with PCD might be uniquely positioned to benefit from antioxidant therapy. Biomarkers for antioxidant efficacy might include an increase in F_ENO_ or nNO and/or a decrease in breath H_2_O_2_. Having these surrogate biomarkers could solve a chronic problem in antioxidant therapy: deciding the modality and dose that are most likely to be effective. Therapeutic options with proven efficacy for patients with PCD are quite limited. 

Note that we have used Derp1 allergen in our study because it belongs to *group 1* allergens, and at least 70% of allergic individuals recognize *group 1* allergens. Derp1 is commonly used in diagnostic tests and in allergy and asthma research. Importantly, Derp1 has bioactivity as a cysteine protease (39, 40). There is a potential role for the cysteine protease activity of Derp1 in this effect. The defective ciliary function would also affect the clearance of other pathogens (bacterial, fungal, and viral) or particulates that may affect activation or expression of pro-oxidant enzymes, such as NADPH oxidase NOX4 (11), NOX 1–2 (41–43), cytochrome P450 enzymes XO (44), and antioxidant enzymes such as SOD (17). Although our evidence suggests that NOX enzyme activity may play a role in airway diseases associated with dysfunctional mucociliary clearance, NOX4 is the only enzyme that has been directly associated with cilia dysfunction (11), and we did not find that expression of NOX 1–4 or of XO was abnormal in our PCD cells (Supplemental Figs. S2–S4). Defective ciliary function leads to activation or increased expression of pro-oxidant enzymes; this leads to NO oxidation and damage to airway epithelial cells. Indeed, this phenomenon may predispose PCD patients to asthma (28) (Fig. 6).

Our study has limitations. We have only done in vitro studies in cells from patients with four representative genetic defects, and three of these—*CCNO, CCDC39,* and *CCDC40*—have recently been identified as possibly associated with more severe disease (27, 45). Future studies on cells from subjects with additional genotypes will be valuable, including those associated with higher nNO. We have not found striking biochemical differences between PCD genotypes, but limitations regarding cell genotype availability over time were such that we were not able to perform all experiments with all cell types. In addition, it may be activity, rather than expression, of oxidant enzymes such as NOX isoforms that may increase NO consumption in the PCD airway; this will require additional study. Additional work is also needed to understand whether antigen stasis-stimulated DUOX1 production also leads directly to downstream effects such as increased IL33 (32), and to better understand the mechanisms by which O_2_^–^ production is increased in PCD. Finally, we have not yet used antioxidants in vivo to determine whether they increase airway NO levels or decrease expired H_2_O_2_ levels.

### Conclusions

In summary, airway cells from patients with PCD do not clear antigens effectively from the luminal surface. This antigen stasis can upregulate oxidant defenses in the airway, consuming NO and potentially injuring the epithelium. Although none of these three observations is, by itself, altogether surprising, we present evidence here to “connect the dots.” This evidence suggests that there may be targetable pathways that could lead to improved management for patients with a variety of PCD genotypes.

## DATA AVAILABILITY

Data will be made available upon reasonable request.

## SUPPLEMENTAL DATA

10.17632/vvsb8xnk67.3 Supplemental Figs. S1–S4: https://doi.org/10.17632/vvsb8xnk67.3.

## GRANTS

This work was supported in part by the NIH Grants P01 HL158507 and P01 HL128192 (to B.G., L.A.S., M.D.D., and N.M.) and HL128370 and HL146601 (to S.L.B.).

## DISCLOSURES

No conflicts of interest, financial or otherwise, are declared by the authors.

## AUTHOR CONTRIBUTIONS

B.G. and N.M. conceived and designed research; L.A.S., M.D.D., I.D., O.G., B.G., and N.M. performed experiments; M.D.D., Y.Z., and N.M. analyzed data; N.M. interpreted results of experiments; L.A.S., M.D.D., and J.S. prepared figures; B.G., A.H., S.L.B., and N.M. edited and revised manuscript; B.G. approved final version of manuscript.

## References

[1] Leigh MW, Horani A, Kinghorn B, O’Connor MG, Zariwala MA, Knowles MR. Primary ciliary dyskinesia (PCD): a genetic disorder of motile cilia. Transl Sci Rare Dis 4: 51–75, 2019. doi:10.3233/TRD-190036. 31572664 PMC6768089

[2] Hannah WB, Seifert BA, Truty R, Zariwala MA, Ameel K, Zhao Y, Nykamp K, Gaston B. The global prevalence and ethnic heterogeneity of primary ciliary dyskinesia gene variants: a genetic database analysis. Lancet Respir Med 10: 459–468, 2022. doi:10.1016/S2213-2600(21)00453-7. 35051411 PMC9064931

[3] Shapiro AJ, Zariwala MA, Ferkol T, Davis SD, Sagel SD, Dell SD, Rosenfeld M, Olivier KN, Milla C, Daniel SJ, Kimple AJ, Manion M, Knowles MR, Leigh MW; Genetic Disorders of Mucociliary Clearance Consortium. Diagnosis, monitoring, and treatment of primary ciliary dyskinesia: PCD foundation consensus recommendations based on state of the art review. Pediatr Pulmonol 51: 115–132, 2016. doi:10.1002/ppul.23304. 26418604 PMC4912005

[4] Noone PG, Leigh MW, Sannuti A, Minnix SL, Carson JL, Hazucha M, Zariwala MA, Knowles MR. Primary ciliary dyskinesia: diagnostic and phenotypic features. Am J Respir Crit Care Med 169: 459–467, 2004. doi:10.1164/rccm.200303-365OC. 14656747

[5] Walker WT, Liew A, Harris A, Cole J, Lucas JS. Upper and lower airway nitric oxide levels in primary ciliary dyskinesia, cystic fibrosis and asthma. Respir Med 107: 380–386, 2013. doi:10.1016/j.rmed.2012.11.021. 23290188

[6] Marozkina N, Bosch J, Cotton C, Smith L, Seckler J, Zaman K, Rehman S, Periasamy A, Gaston H, Altawallbeh G, Davis M, Jones DR, Schilz R, Randell SH, Gaston B. Cyclic compression increases F508 Del CFTR expression in ciliated human airway epithelium. Am J Physiol Lung Cell Mol Physiol 317: L247–L258, 2019. doi:10.1152/ajplung.00020.2019. 31116581 PMC6734384

[7] Marozkina NV, Gaston B. Nitrogen chemistry and lung physiology. Annu Rev Physiol 77: 431–452, 2015. doi:10.1146/annurev-physiol-021113-170352. 25668023

[8] van der Vliet A, Eiserich JP, Shigenaga MK, Cross CE. Reactive nitrogen species and tyrosine nitration in the respiratory tract: epiphenomena or a pathobiologic mechanism of disease? Am J Respir Crit Care Med 160: 1–9, 1999. doi:10.1164/ajrccm.160.1.9807044. 10390372

[9] Fulcher ML, Randell SH. Human nasal and tracheo-bronchial respiratory epithelial cell culture. Methods Mol Biol 945: 109–121, 2013. doi:10.1007/978-1-62703-125-7_8. 23097104

[10] Price ME, Sisson JH. Redox regulation of motile cilia in airway disease. Redox Biol 27: 101146, 2019. doi:10.1016/j.redox.2019.101146. 30833143 PMC6859573

[11] Wan WY, Hollins F, Haste L, Woodman L, Hirst RA, Bolton S, Gomez E, Sutcliffe A, Desai D, Chachi L, Mistry V, Szyndralewiez C, Wardlaw A, Saunders R, O'Callaghan C, Andrew PW, Brightling CE. NADPH oxidase-4 overexpression is associated with epithelial ciliary dysfunction in neutrophilic asthma. Chest 149: 1445–1459, 2016. doi:10.1016/j.chest.2016.01.024. 26836936 PMC4893823

[12] Gray D, Lissi E, Heicklen J. Reaction of hydrogen peroxide with nitrogen dioxide and nitric oxide. J Phys Chem 76: 1919–1924, 1972. doi:10.1021/j100658a001.

[13] Haddad IY, Pataki G, Hu P, Galliani C, Beckman JS, Matalon S. Quantitation of nitrotyrosine levels in lung sections of patients and animals with acute lung injury. J Clin Invest 94: 2407–2413, 1994. doi:10.1172/JCI117607. 7989597 PMC330071

[14] van der Vliet A, Danyal K, Heppner DE. Dual oxidase: a novel therapeutic target in allergic disease. Br J Pharmacol 175: 1401–1418, 2018. doi:10.1111/bph.14158. 29405261 PMC5900994

[15] Hristova M, Habibovic A, Veith C, Janssen-Heininger YM, Dixon AE, Geiszt M, van der Vliet A. Airway epithelial dual oxidase 1 mediates allergen-induced IL-33 secretion and activation of type 2 immune responses. J Allergy Clin Immunol 137: 1545–1556.e11, 2016. doi:10.1016/j.jaci.2015.10.003. 26597162 PMC4860024

[16] Comhair SA, Xu W, Ghosh S, Thunnissen FB, Almasan A, Calhoun WJ, Janocha AJ, Zheng L, Hazen SL, Erzurum SC. Superoxide dismutase inactivation in pathophysiology of asthmatic airway remodeling and reactivity. Am J Pathol 166: 663–674, 2005. doi:10.1016/S0002-9440(10)62288-2. 15743779 PMC1602353

[17] Comhair SA, Bhathena PR, Dweik RA, Kavuru M, Erzurum SC. Rapid loss of superoxide dismutase activity during antigen-induced asthmatic response. Lancet 355: 624, 2000. doi:10.1016/S0140-6736(99)04736-4. 10696986

[18] Wallmeier J, Al-Mutairi DA, Chen CT, Loges NT, Pennekamp P, Menchen T, Ma L, Shamseldin HE, Olbrich H, Dougherty GW, Werner C, Alsabah BH, Köhler G, Jaspers M, Boon M, Griese M, Schmitt-Grohé S, Zimmermann T, Koerner-Rettberg C, Horak E, Kintner C, Alkuraya FS, Omran H. Mutations in CCNO result in congenital mucociliary clearance disorder with reduced generation of multiple motile cilia. Nat Genet 46: 646–651, 2014. doi:10.1038/ng.2961. 24747639

[19] Johnson MA, Macdonald TL, Mannick JB, Conaway MR, Gaston B. Accelerated s-nitrosothiol breakdown by amyotrophic lateral sclerosis mutant copper, zinc-superoxide dismutase. J Biol Chem 276: 39872–39878, 2001. doi:10.1074/jbc.M102781200. 11518706

[20] Ismail HM, Scapozza L, Ruegg UT, Dorchies OM. Diapocynin, a dimer of the NADPH oxidase inhibitor apocynin, reduces ROS production and prevents force loss in eccentrically contracting dystrophic muscle. PLoS One 9: e110708, 2014. doi:10.1371/journal.pone.0110708. 25329652 PMC4201587

[21] Stefanska J, Pawliczak R. Apocynin: molecular aptitudes. Mediators Inflamm 2008: 106507, 2008. doi:10.1155/2008/106507. 19096513 PMC2593395

[22] Asano K, Chee CB, Gaston B, Lilly CM, Gerard C, Drazen JM, Stamler JS. Constitutive and inducible nitric oxide synthase gene expression, regulation, and activity in human lung epithelial cells. Proc Natl Acad Sci USA 91: 10089–10093, 1994. doi:10.1073/pnas.91.21.10089. 7524082 PMC44963

[23] Gow A, Doctor A, Mannick J, Gaston B. S-Nitrosothiol measurements in biological systems. J Chromatogr B Analyt Technol Biomed Life Sci 851: 140–151, 2007. doi:10.1016/j.jchromb.2007.01.052. 17379583 PMC1949323

[24] Dweik RA, Comhair SA, Gaston B, Thunnissen FB, Farver C, Thomassen MJ, Kavuru M, Hammel J, Abu-Soud HM, Erzurum SC. NO chemical events in the human airway during the immediate and late antigen-induced asthmatic response. Proc Natl Acad Sci USA 98: 2622–2627, 2001. doi:10.1073/pnas.051629498. 11226289 PMC30188

[25] Horváth I, Barnes PJ, Loukides S, Sterk PJ, Högman M, Olin AC, , et al. A European Respiratory Society technical standard: exhaled biomarkers in lung disease. Eur Respir J 49: 1600965, 2017. doi:10.1183/13993003.00965-2016. 28446552

[26] Schultz R, Elenius V, Lukkarinen H, Saarela T. Two novel mutations in the DNAH11 gene in primary ciliary dyskinesia (CILD7) with considerable variety in the clinical and beating cilia phenotype. BMC Med Genet 21: 237–237, 2020. doi:10.1186/s12881-020-01171-2. 33243178 PMC7690114

[27] Davis SD, Rosenfeld M, Lee HS, Ferkol TW, Sagel SD, Dell SD, Milla C, Pittman JE, Shapiro AJ, Sullivan KM, Nykamp KR, Krischer JP, Zariwala MA, Knowles MR, Leigh MW. Primary ciliary dyskinesia: longitudinal study of lung disease by ultrastructure defect and genotype. Am J Respir Crit Care Med 199: 190–198, 2019. doi:10.1164/rccm.201803-0548OC. 30067075 PMC6353004

[28] Levine H, Bar-On O, Nir V, West N, Dizitzer Y, Mussaffi H, Prais D. Reversible bronchial obstruction in primary ciliary dyskinesia. J Clin Med 11: 6791, 2022. doi:10.3390/jcm11226791. 36431268 PMC9699262

[29] Piacentini GL, Bodini A, Peroni D, Rigotti E, Pigozzi R, Pradal U, Boner AL. Nasal nitric oxide for early diagnosis of primary ciliary dyskinesia: practical issues in children. Respir Med 102: 541–547, 2008. doi:10.1016/j.rmed.2007.11.013. 18187313

[30] Kettle AJ, Chan T, Osberg I, Senthilmohan R, Chapman AL, Mocatta TJ, Wagener JS. Myeloperoxidase and protein oxidation in the airways of young children with cystic fibrosis. Am J Respir Crit Care Med 170: 1317–1323, 2004. doi:10.1164/rccm.200311-1516OC. 15466253

[31] Wedes SH, Wu W, Comhair SA, McDowell KM, DiDonato JA, Erzurum SC, Hazen SL. Urinary bromotyrosine measures asthma control and predicts asthma exacerbations in children. J Pediatr 159: 248–255.e1, 2011. doi:10.1016/j.jpeds.2011.01.029. 21392781 PMC3354913

[32] Cook DP, Thomas CM, Wu AY, Rusznak M, Zhang J, Zhou W, Cephus JY, Gibson-Corley KN, Polosukhin VV, Norlander AE, Newcomb DC, Stoltz DA, Peebles RS Jr. Cystic fibrosis reprograms airway epithelial IL-33 release and licenses IL-33-dependent inflammation. Am J Respir Crit Care Med 207: 1486–1497, 2023. doi:10.1164/rccm.202211-2096OC. 36952660 PMC10263140

[33] Mendelsohn L, Wijers C, Gupta R, Marozkina N, Li C, Gaston B. A novel, noninvasive assay shows that distal airway oxygen tension is low in cystic fibrosis, but not in primary ciliary dyskinesia. Pediatr Pulmonol 54: 27–32, 2019. doi:10.1002/ppul.24192. 30485726

[34] Worlitzsch D, Tarran R, Ulrich M, Schwab U, Cekici A, Meyer KC, Birrer P, Bellon G, Berger J, Weiss T, Botzenhart K, Yankaskas JR, Randell S, Boucher RC, Döring G. Effects of reduced mucus oxygen concentration in airway Pseudomonas infections of cystic fibrosis patients. J Clin Invest 109: 317–325, 2002. doi:10.1172/JCI13870. 11827991 PMC150856

[35] Marozkina N, Smith L, Zhao Y, Zein J, Chmiel JF, Kim J, Kiselar J, Davis MD, Cunningham RS, Randell SH, Gaston B. Somatic cell hemoglobin modulates nitrogen oxide metabolism in the human airway epithelium. Sci Rep 11: 15498, 2021. doi:10.1038/s41598-021-94782-5. 34326365 PMC8322277

[36] Gaston B, Ratjen F, Vaughan JW, Malhotra NR, Canady RG, Snyder AH, Hunt JF, Gaertig S, Goldberg JB. Nitrogen redox balance in the cystic fibrosis airway: effects of antipseudomonal therapy. Am J Respir Crit Care Med 165: 387–390, 2002. doi:10.1164/ajrccm.165.3.2106006. 11818326

[37] Wijers CD, Chmiel JF, Gaston BM. Bacterial infections in patients with primary ciliary dyskinesia: comparison with cystic fibrosis. Chron Respir Dis 14: 392–406, 2017. doi:10.1177/1479972317694621. 29081265 PMC5729729

[38] Marozkina NV, Yemen S, Borowitz M, Liu L, Plapp M, Sun F, Islam R, Erdmann-Gilmore P, Townsend RR, Lichti CF, Mantri S, Clapp PW, Randell SH, Gaston B, Zaman K. Hsp 70/Hsp 90 organizing protein as a nitrosylation target in cystic fibrosis therapy. Proc Natl Acad Sci USA 107: 11393–11398, 2010. doi:10.1073/pnas.0909128107. 20534503 PMC2895117

[39] Wan H, Winton HL, Soeller C, Tovey ER, Gruenert DC, Thompson PJ, Stewart GA, Taylor GW, Garrod DR, Cannell MB, Robinson C. Der p 1 facilitates transepithelial allergen delivery by disruption of tight junctions. J Clin Invest 104: 123–133, 1999. doi:10.1172/JCI5844. 10393706 PMC408401

[40] Herbert CA, King CM, Ring PC, Holgate ST, Stewart GA, Thompson PJ, Robinson C. Augmentation of permeability in the bronchial epithelium by the house dust mite allergen Der p1. Am J Respir Cell Mol Biol 12: 369–378, 1995. doi:10.1165/ajrcmb.12.4.7695916. 7695916

[41] Fink K, Duval A, Martel A, Soucy-Faulkner A, Grandvaux N. Dual role of NOX2 in respiratory syncytial virus- and sendai virus-induced activation of NF-kappaB in airway epithelial cells. J Immunol 180: 6911–6922, 2008. doi:10.4049/jimmunol.180.10.6911. 18453612

[42] Heppner DE, Hristova M, Dustin CM, Danyal K, Habibovic A, van der Vliet A. The NADPH oxidases DUOX1 and NOX2 play distinct roles in redox regulation of epidermal growth factor receptor signaling. J Biol Chem 291: 23282–23293, 2016. doi:10.1074/jbc.M116.749028. 27650496 PMC5087744

[43] Krick S, Wang J, St-Pierre M, Gonzalez C, Dahl G, Salathe M. Dual oxidase 2 (Duox2) regulates pannexin 1-mediated ATP release in primary human airway epithelial cells via changes in intracellular pH and not H_2_O_2_ production. J Biol Chem 291: 6423–6432, 2016. doi:10.1074/jbc.M115.664854. 26823467 PMC4813552

[44] Feldman C, Anderson R, Kanthakumar K, Vargas A, Cole PJ, Wilson R. Oxidant-mediated ciliary dysfunction in human respiratory epithelium. Free Radic Biol Med 17: 1–10, 1994. doi:10.1016/0891-5849(94)90002-7. 7959161

[45] Henriques AR, Constant C, Descalço A, Pinto A, Moura Nunes J, Sampaio P, Lopes SS, Pereira L, Bandeira T. Primary ciliary dyskinesia due to CCNO mutations-A genotype-phenotype correlation contribution. Pediatr Pulmonol 56: 2776–2779, 2021. doi:10.1002/ppul.25440. 34102041

